# Inter-rater reliability assessment for the new-born screening quality assurance

**DOI:** 10.11613/BM.2022.030901

**Published:** 2022-08-05

**Authors:** Leonor Guiñón, Anna Soler, Rosa María López, Sonia Pajares, José Manuel González de Aledo, Ana Argudo-Ramírez, José Luis Marín, Judit García-Villoria, Ángeles Sahuquillo, Luisa Alvarez

**Affiliations:** 1 Quality Department, Biomedical Diagnostic Center, Hospital Clínic of Barcelona, Barcelona, Spain; 2 Quality Department, Laboratories, Hospital de la Santa Creu i Sant Pau of Barcelona, Barcelona, Spain; 3 Biochemistry Department, Hospital de la Santa Creu i Sant Pau of Barcelona, Barcelona, Spain; 4 Division of Inborn Errors of Metabolism, Biochemistry and Molecular Genetics Department, Biomedical Diagnostic Center, Hospital Clínic of Barcelona, IDIBAPS, Barcelona Spain; 5 Spain Center for Biomedical Research Network on Rare Diseases (CIBERER), Madrid, Spain; 6 Section of Analytical Chemistry, Faculty of Chemistry, University of Barcelona, Barcelona, Spain

**Keywords:** accreditation, extra-analytical phase, new-born screening, quality control

## Abstract

**Introduction:**

To ensure the quality of the new-born screening (NBS), our laboratory reviewed the analytical procedure to detect subjective steps that may represent a risk to the patient. Two subjective activities were identified in the extra-analytical phases: the classification of dried blood spots (DBS) according to their quality and the assignment of haemoglobin patterns. To keep these activities under control, inter-rater studies were implemented. This study aimed to evaluate the inter-rater reliability and the effectiveness of the measures taken to improve the agreement between observers, to assure NBS results’ quality.

**Materials and methods:**

Dried blood spots specimens were used for the inter-rater studies. Ten studies were performed to assess DBS quality classification, and four to assess the assignment of haemoglobin patterns. Krippendorff’s alpha test was used to estimate inter-rater reliability. Causes were investigated when alpha values were below 0.80.

**Results:**

For both activities, the reliability obtained in the first studies was inadequate. After investigation, we detected that the criterion to classify a DBS as scant was not consolidated, and also a lack of consensus on whether or not to report Bart’s haemoglobin depending on its percentage. Alpha estimates became higher once the training was reinforced and a consensus about the appropriate criteria to be applied was reached.

**Conclusion:**

Inter-rater reliability assessment helped us to ensure the quality of subjective activities that could add variability to NBS results. Furthermore, the evolution of the alpha value over time allowed us to verify the effectiveness of the measures adopted.

## Introduction

It is estimated that 70 percent of clinical decisions are based on laboratory tests’ results (*1*). For this reason, laboratories must ensure the reliability of results, since they could directly affect patient safety. Clinical laboratories follow different strategies to assure the quality of reported results. One of the essential tools is participation in external quality assessment and proficiency testing schemes. However, these programs generally do not address all aspects of the analytical procedure that can affect results’ reliability. Extra-analytical steps are usually not included, whereas the majority of errors occur in the pre-analytical (68.2%) or post-analytical phases (18.5%) (*2*). In this regard, the International Organization for Standardization (ISO) 15189:2012 standard (subclause 5.6.1) requires the implementation of appropriate pre- and post-examination processes for the quality assurance of examination results (*3*). Therefore, to add appropriate control measures a thorough revision of the extra-analytical phases must be performed to detect those aspects that may represent a risk to the patient.

New-born screening (NBS) allows the early detection of congenital diseases and it is intended to initiate early treatment to reduce morbidity and mortality. Laboratories receive dried blood spots (DBS) for testing, that is a specimen of blood collected onto a particular filter paper on which printed circles indicate the area to be filled. The panel of screened diseases varies depending on the country. The Catalan NBS program (in Spain) includes the screening of amino acidemias, organic acidemias, fatty acid oxidation disorders, congenital hypothyroidism, cystic fibrosis, sickle cell disease, and severe combined immunodeficiency disease. As in any other screening program, the existence of false positive and negative results is assumed. These false results may be due not only to the biological variability of the measurands, the analytical interferences (*e.g.* caused by drugs or body creams among others), or the analytical performance of technology, but also to failures in other aspects of the NBS program that depend on the decisions taken by the personnel involved in the different phases of the procedure.

Our laboratory reviewed the whole procedure of the NBS to ensure that we provided the highest-quality results. We performed a risk assessment to detect if it was necessary to add control measures to the laboratory’s quality control plan. As a result, we identified two potential failures that may represent a risk to the patient and that were not addressed by the quality control plan established. The first one was the classification of the DBS specimen’s quality performed in the pre-analytical phase. Despite the existence of sampling guidelines, it is common for NBS laboratories to receive DBS under non-optimal conditions (*4*). Dried blood spots quality is evaluated by visual inspection; therefore, the decision of acceptance or rejection of specimens can differ between observers, and this decision can lead to false positive or negative results (*5*). The second one was the assignment of haemoglobin (Hb) patterns carried out in the post-analytical phase from the visualization of electrophoreticgrams. In Europe, only a few countries screen for haemoglobinopathies, and only sickle cell disease is included in the screening panels (*6*). However, the detection of other haemoglobinopathies of interest is also possible. In this regard, the European Consensus issued the recommendation of reporting beta thalassaemia in 2017 (*7*). In any case, observers’ subjectivity may lead to a wrong result in the screening for haemoglobinopathies. Consequently, as both activities could present subjectivity between observers, inter-rater studies were implemented to keep these steps under control.

In this context, this study aimed to evaluate the inter-rater reliability to assure the quality of NBS results, as well as to assess the effectiveness of the measures taken to improve the agreement between observers.

## Materials and methods

### Materials

Blood from a heel prick was collected onto Whatman 903 paper (Whatman International Ltd, Chalfont St Giles, United Kingdom) for NBS. Fifty DBS were used to perform ten inter-rater studies to assess the agreement on the classification of the specimen’s quality in the period between 2015 and 2020. Of the selected DBS, 22 had an acceptable quality and 28 were unacceptable (6 scant and 22 non-optimal specimens). Twenty-five DBS were used to conduct four inter-rater studies to assess the agreement on the assignment of Hb patterns between 2017 and 2020. For these studies, the following cases were selected: 2 unaffected, 15 affected (3 beta thalassaemia major, 7 sickle cell disease, 3 alpha thalassaemia, 1 Hb E disease and 1 Hb D disease) and 8 carriers of Hb variants.

Specific data about the samples included in each study and the number of observers who participated are shown in Table 1.

**Table 1:** Number of participants and samples included in the inter-rater studies

**Dried blood spots’ quality classification**				
**Study no./Year**	**Observers**	**Samples**	**Samples included**	
			**N**	**Classification**
1/2015	8	5	2	Acceptable
			2	Scant
			1	Non-optimal
2/2015	7	5	3	Acceptable
			2	Non-optimal
3/2016	6	5	3	Acceptable
			2	Non-optimal
4/2017	7	5	4	Acceptable
			1	Non-optimal
5/2017	7	5	3	Acceptable
			2	Non-optimal
6/2018	7	5	1	Acceptable
			1	Scant
			3	Non-optimal
7/2018	7	5	2	Acceptable
			1	Scant
			2	Non-optimal
8/2019	7	5	1	Acceptable
			1	Scant
			3	Non-optimal
9/2020	7	5	2	Acceptable
			1	Scant
			2	Non-optimal
10/2020	7	5	1	Acceptable
			4	Non-optimal
**Haemoglobin pattern assignment**				
**Study no./Year**	**Observers**	**Samples**	**Samples included**	
			**N**	**Hb pattern**
1/2017	4	10	1	FA*
			1	F
			1	FS
			1	FCS
			1	FA Bart’s
			5	FAX
2/2018	4	5	1	FA*
			1	F
			1	FS
			1	FAC
			1	FAX
**Study no./Year**	**Observers**	**Samples**	**Samples included**	
			**N**	**Hb pattern**
3/2019	5	5	1	FS
			1	FE
			1	FA Bart’s
			1	FAX
			1	FSX
4/2020	5	5	1	F
			1	FD
			1	FA Bart’s
			2	FSA
*Normal haemoglobin pattern in new-borns. F - foetal haemoglobin. A - adult haemoglobin. Bart’s - Bart’s haemoglobin. S, C, D, E - β-chain haemoglobin variants S, C, D, and E. X - rare haemoglobin variants.				

### Methods

The inter-rater studies performed to assess the agreement on the classification of DBS specimen’s quality were carried out by visual inspection before the punching of the spot. Specimens were classified as acceptable, scant, or non-optimal according to the following criteria:

- Acceptable: Blood filled all spots and they were evenly saturated.
- Unacceptable:
  1. Scant: Blood transferred to the back side of the paper but it was not enough to perform all tests.
  2. Non-optimal: a) Insufficient: Blood did not transfer to the back side of the paper; b) Compressed: Blood filled the spots but they were not saturated; c) Over-impregnated: Blood applied to both sides of the paper; d) Multispotted: Multiple small bloodspots to make one larger.

The inter-rater studies conducted to assess the agreement on the assignment of Hb patterns were carried out by visual inspection of the electrophoreticgrams obtained by capillary electrophoresis. All tests were run on Capillarys 2 Neonat Fast (Sebia, Lisses, France). The criteria followed for reporting the pattern was:

- Hb fractions were sorted by decreasing concentration (*i.e.* a report of FA pattern indicates that the concentration of HbF is higher than that of HbA, which is the normal pattern in new-borns).
- Rare Hb variants (*i.e.* other than S, C, D, E, and Bart’s) were coded as X.

### Statistical analysis

Krippendorff’s alpha test was used to estimate the inter-rater reliability. A 10,000 bootstrap samples were taken to produce confidence intervals at the 95% level. Estimates of the probability that the true alpha value was less than 0.80 were also obtained (*8*). Statistical analysis was performed using SPSS v19.

The alpha estimates were interpreted following Krippendorff’s suggestions (Table 2) (*9*). Alpha values close to 1 indicated perfect agreement. Below 0.80 but above 0.67 indicated low reliability. An alpha below 0.67 indicated a really low inter-rater reliability. When alpha values were below 0.80, causes were investigated in order to implement corrective measures.

**Table 2:** Interpretation of Krippendorff’s alpha

**Value of alpha**	**Level of agreement**
1	Perfect
> 0.80	Good
0.67-0.80	Low
< 0.67	Really low
0	None

## Results

For both activities, the results showed that the inter-rater reliability of the first studies performed was inadequate (Table 3). The first alpha estimate for the classification of DBS quality was far lower (0.59) than the one obtained for the assignment of Hb patterns, which was close to achieving a good level of agreement (0.79).

**Table 3:** Inter-rater reliability of the studies performed to assess the classification of dried blood spot’s quality and the assignment of haemoglobin patterns

**Dried blood spots’ quality classification**			
**Study no. / Year**	**Alpha**	**95% CI**	**q (%)**
1/2015	0.59	0.47-0.70	99.99
2/2015	0.88	0.78-0.96	3.76
3/2016	0.86	0.72-0.97	12.23
4/2017	1.00	nc	nc
5/2017	1.00	nc	nc
6/2018	1.00	nc	nc
7/2018	0.77	0.67-0.87	64.96
8/2019	0.85	0.75-0.92	19.66
9/2020	1.00	nc	nc
10/2020	1.00	nc	nc
**Haemoglobin pattern assignment**			
1/2017	0.79	0.67-0.90	55.39
2/2018	0.88	0.76-1.00	7.37
3/2019	0.91	0.81-0.98	1.63
4/2020	0.90	0.79-0.97	4.30
95% CI – 95% confidence interval. q - probability that the true alpha value was below 0.8. nc - not calculable.			

When investigating the causes behind these low alpha values, the lack of unified criteria between the personnel in certain aspects was revealed. Firstly, in the classification of the DBS quality, disagreement between observers in classifying two types of unacceptable specimens was observed: a DBS that had insufficient blood on the spots (*i.e.* the blood did not transfer to the back side of the paper); and, a DBS that was acceptable to perform some tests but was not enough to complete all screening tests (*i.e.* few spots filled with blood and evenly saturated). No differences between observers in the classification of other unacceptable DBS specimens (*i.e*. multispotted, over-impregnated or compressed bloodspots) were identified. Secondly, in the assignment of Hb patterns, disagreement among the observers in deciding whether or not to report the presence of Bart’s Hb depending on the quantity detected was observed. No discrepancies in the assignment of other pathological Hb patterns or the normal Hb pattern (*i.e.* FA) were found.

Once the technicians performed a specific training with insufficient and scant DBS, and the laboratory specialists met a consensus about Bart’s Hb, the alpha estimates became higher than 0.80 in the majority of the studies performed, showing strong inter-rater reliability. Only in one study carried out to assess the DBS’s quality classification in 2018 an alpha value a bit lower than 0.80 was obtained, with a maximum estimated 64.96 percent chance of alpha failing to exceed 0.80. When investigating the causes, we observed that most of the disagreements belonged to recently hired technical staff, which classified some acceptable DBS specimens as non-optimal.

## Discussion

The interpretation of qualitative results presents greater subjectivity than that of quantitative results. Therefore, laboratories must apply the tools at their disposal to make interpretations as objective and homogeneous as possible. In our particular case, the assessment of the inter-rater reliability helped us to ensure the quality of those extra-analytical activities that depended, in whole or in part, on visual examination and that could affect the NBS results.

The root-cause analysis performed to identify the causes of the poor reliability in the first inter-rater study in the classification of DBS quality revealed the need to re-educate the technical staff to clarify the differences between a scant sample and an insufficient sample. Based on this classification, the decision of accepting or rejecting the specimen is taken, leading to different consequences. On the one hand, the rejection of a DBS specimen that is acceptable to perform a portion of tests can lead to a delay in the detection of disease, since it implies requesting a new sample for testing. On the other hand, acceptance of a DBS specimen with insufficient blood may lead to a false negative result for some measurands (*5*). In both cases, early detection of the disease is not carried out, thus endangering the life of the new-born. In this regard, it should be noted that our laboratory performs testing on DBS specimens with unacceptable quality in order to detect possible positive results, assuming that false positive (*e.g.* multispotted samples) or false negative results (*e.g.* compressed bloodspots) can be obtained. However, the final results report is not released until a repeat screening on an additional requested specimen is performed, as long as the new specimen has an acceptable quality. The root-cause analysis performed after the seventh inter-rater study showed the need to enhance training in the classification of DBS specimens for the new staff, to provide them with the expertise required.

The root-cause analysis performed after the first inter-rater study for the assignment of Hb patterns revealed discrepancies between the laboratory specialists when reporting Bart’s Hb. Some participants reported Bart’s Hb only when the fraction detected was higher than 15%. Other participants reported always Bart’s Hb regardless of the percentage. Based on the fact that low percentages do not require further testing or referral to a haematologist, for the next inter-rater studies, a consensus on reporting Bart’s Hb only when the fraction was higher than 15% was reached (*10*).

The evolution of the alpha value over time allowed us to verify the effectiveness of the corrective measures implemented. For both activities, a better level of agreement between observers was achieved in the subsequent studies. Despite the rapid increase of the alpha value observed in the second inter-rater studies performed, continuous training and education of the laboratory personnel was required to sustain a high level of agreement. Thus, we established to conduct inter-rater studies at least once a year; and, additionally, when recruiting new staff or after a long-term leave.

Other authors have assessed the inter-rater reliability in order to implement actions to minimize the effect of the human factor in laboratory results and have even informed clinicians about the degree of reliability attained (*11*, *12*). Some of them have also used Krippendorff’s alpha test for the assessment; but, to our knowledge, no previous studies have assessed whether inter-rater variability could affect NBS results (*13*).

In conclusion we can say that conducting inter-rater exercises is essential to control subjective activities that can lead to an error or delay in diagnosis. Their implementation in our laboratory helped us to detect controversial points in the decision making and to focus corrective measures to standardize important criteria. The assessment of the inter-rater reliability allowed us to verify if the measures taken were and remain effective over time, thus boosting the quality assurance of NBS. Moreover, standardization of subjective activities helped us to accomplish the quality assurance requirements of test results described in the ISO 15189:2012 standard.
